# Identification of absolute geometries of *cis* and *trans* molecular isomers by Coulomb Explosion Imaging

**DOI:** 10.1038/srep38202

**Published:** 2016-12-02

**Authors:** Utuq Ablikim, Cédric Bomme, Hui Xiong, Evgeny Savelyev, Razib Obaid, Balram Kaderiya, Sven Augustin, Kirsten Schnorr, Ileana Dumitriu, Timur Osipov, René Bilodeau, David Kilcoyne, Vinod Kumarappan, Artem Rudenko, Nora Berrah, Daniel Rolles

**Affiliations:** 1 J. R. Macdonald Laboratory, Physics Dept., Kansas State University, Manhattan, KS 66506, USA; 2 Deutsches Elektronen-Synchrotron (DESY), 22607, Hamburg, Germany; 3 Department of Physics, University of Connecticut, Storrs, CT 06269, USA; 4 Max Planck Institute for Nuclear Physics, 69117 Heidelberg, Germany; 5 Hobart and William Smith Colleges, Geneva, NY 14456, USA; 6 Department of Chemistry, University of California, Berkeley, CA 94720, USA; 7 SLAC National Accelerator Laboratory, Menlo Park, CA 94025, USA; 8 Advanced Light Source, Lawrence Berkeley National Laboratory, Berkeley, CA 94720, USA

## Abstract

An experimental route to identify and separate geometric isomers by means of coincident Coulomb explosion imaging is presented, allowing isomer-resolved photoionization studies on isomerically mixed samples. We demonstrate the technique on *cis/trans* 1,2-dibromoethene (C_2_H_2_Br_2_). The momentum correlation between the bromine ions in a three-body fragmentation process induced by bromine 3*d* inner-shell photoionization is used to identify the *cis* and *trans* structures of the isomers. The experimentally determined momentum correlations and the isomer-resolved fragment-ion kinetic energies are matched closely by a classical Coulomb explosion model.

Isomers, i.e. molecules with the same chemical formula but different geometric structures, play an important role in many biological processes^1^,^2^,^3^,^4^,^5^,^6^. For example, our vision requires a protein called rhodopsin, which inter-converts between different geometric structures (i.e. isomers) during the absorption of light^7^. Another example is the green fluorescent protein, which is used to investigate protein trafficking inside living cells and which changes configuration from its well-ordered *cis* isomer to a highly disordered *trans* isomer^8^ upon absorption of light in the blue to ultraviolet range.

Despite containing the same atomic constituents, isomers can have very different physical, chemical, and biological properties. Therefore, it is of particular interest to experimentally distinguish isomers in order to investigate isomer-specific reactions and, specifically, to study the interconversion between different isomers in time-resolved experiments^9^. A prototypical example is the isomerization between acetylene (HCCH) and vinylidene (H_2_CC) via hydrogen migration, which has been studied, e.g. using synchrotron radiation^10^ and by femtosecond time-resolved experiments with intense, ultrashort optical^11^,^12^ and XUV or X-ray pulses^13^,^14^,^15^. All of these experiments have employed the Coulomb explosion coincidence momentum imaging technique^16^,^17^,^18^,^19^, in which all charged fragments that are created when the multiply charged parent ion breaks up (“*explodes*”) are measured in coincidence and their momentum vectors are used to extract information about the dynamical change in the geometry of the parent molecule. Recently, this technique was also used to separate two enantiomers in a racemic mixture of the chiral molecules CHIClF and CHBrClF by measuring five-fold coincidences after strong-field laser ionization^20^,^21^, and the related beam-foil Coulomb explosion method was used to study the absolute configuration of trans-2,3-dideuterooxirane (C_2_H_2_D_2_O)^22^.

Here, we focus on geometric isomers, which have the same bond structure but different positioning of atoms or functional groups in space. Earlier work on the Coulomb explosion of geometric isomers using intense laser pulses found different fragment ion angular distributions, but since those experiments were not performed using coincidence momentum imaging, the data could not be used to identify the geometric structure of the isomers^23^. We present an experimental route to efficiently identify geometric isomers, namely *cis* and *trans* 1,2-dibromoethene (C_2_H_2_Br_2_) in a mixed sample, and demonstrate that by studying the three-body fragmentation of the molecule via Coulomb explosion, one can determine the original molecular structure. In particular, our methodology is directly applicable to time-resolved pump-probe experiments studying the *cis*-*trans* isomerization using either strong-field or inner-shell ionization as a probe since it allows performing an *in-situ* measurement of the isomer ratio of an isomerically mixed sample.

## Results and Discussion

**Figure 1:**
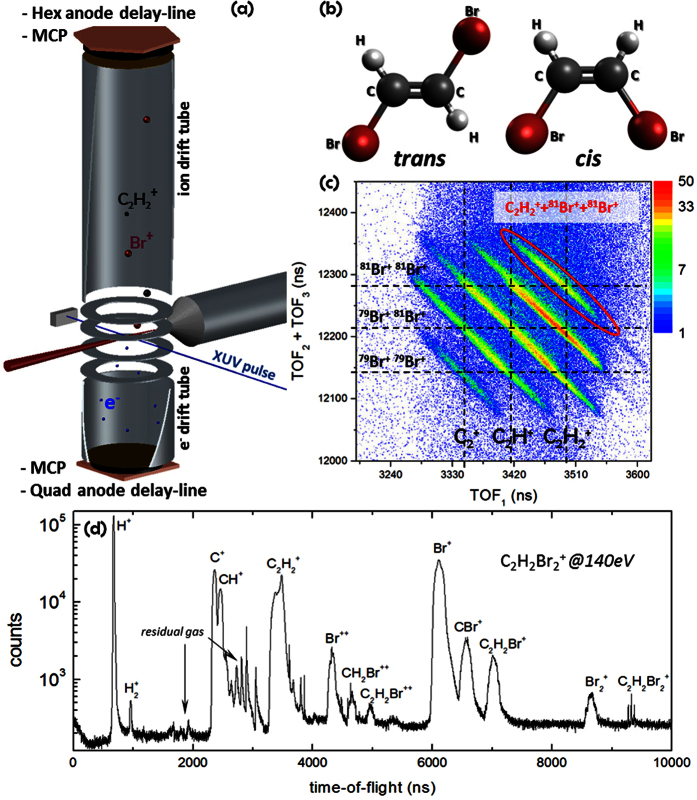
(**a**) Schematic of the experimental setup including a supersonic molecular beam and a double-sided VMI spectrometer with time- and position-sensitive delay line detectors. The spacing between the spectrometer electrodes is 15 mm, and the total distance between the interaction region and the detector is 398 mm for the ions and 112 mm for the electrons. (**b**) Molecular structure of *cis* and *trans* 1,2-dibromoethene. (**c**) Yield of the C_2_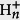 + Br^+^ + Br^+^ (*n* = 0, 1, 2) coincidence channel shown in a photoion-photoion-photoion coincidence (PIPIPICO) plot as a function of the time of flight (TOF) of the first hit versus the sum of the TOFs for the second and third hits. (**d**) Ion time-of-flight mass spectrum of all ions generated in the X-ray induced ionization and fragmentation of 1,2-dibromoethene at 140 eV photon energy.

The experiment was conducted at beamline 10.0.1.3 of the Advanced Light Source (ALS) at Lawrence Berkeley National Laboratory. We used a double-sided velocity map imaging (VMI) setup, where ions and electrons are measured in coincidence, as shown in Fig. 1(a).

The chemical sample used in the experiment was a mixture of *cis* and *trans* isomers of 1,2-dibromoethene. In the *cis* isomer, the two bromine atoms, each attached to one of the two carbon atoms, are pointing in the same hemisphere with respect to the C=C double bond, while they are across from each other in the *trans* isomer (see Fig. 1(b)). The double bond between the carbon atoms restricts the inter-conversion from one isomer to the other such that the two isomers are stable at room temperature.

For initiating the photoionization and fragmentation of the molecule, we chose a photon energy of 140 eV (bandwidth ±20 meV), which is 63 eV above the binding energy of the bromine 3d inner-shell electrons in 1,2-dibromoethene^24^. Absorption of a 140-eV photon thus leads predominantly to 3*d* inner-shell ionization followed by Auger decay that typically ejects one or two additional electrons. Depending on the specific relaxation pathway, the resulting doubly or triply charged molecular cation typically breaks up into several charged and/or neutral fragments (see Fig. 1(d)). Here, we concentrate on the three-body fragmentation channel C_2_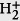 + Br^+^ + Br^+^, for which we measure the time of flight and hit positions of all three fragments in coincidence.

While we are not aware of an electron-electron coincidence measurements on C_2_H_2_Br_2_, we assume that similar to the case of 3d photoionization of Kr, most of the triply charged final states are reached via sequential emission of two Auger electrons in an Auger cascade^25^. In Kr, the Auger lifetimes are 8 fs for the first step and 20 fs for the second step^26^, and we expect the lifetimes to be of the same order in C_2_H_2_Br_2_.

In the zoomed-in triple-ion coincidence plot in Fig. 1(c), the three-body fragmentation channel C_2_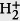 + Br^+^ + Br^+^ appears as sharp diagonal lines due to momentum conservation during the breakup process. In this case, a total of nine diagonal lines are present corresponding to the C_2_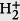 + Br^+^ + Br^+^, C_2_H^+^ + Br^+^ + Br^+^, and 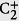 + Br^+^ + Br^+^ channels with different combinations of the two stable bromine isotopes, ^79^Br and ^81^Br, which have approximately equal natural abundance. Since several of these lines are overlapping, we selected only the region containing the C_2_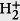 + ^81^Br^+^ + ^81^Br^+^ channel for further analysis since this channel is clearly separated.

In order to reconstruct the ion momenta from the measured time of flight and hit positions of each ion, we used the SIMION software package^27^ to construct empirical formulas connecting the hit position with the momentum vector components parallel to the detector, P_*x*_ and P_*v*_, and the time-of-flight spread of each ion with the momentum component along spectrometer axis, P_*z*_. Using this procedure, the momentum sum of all fragments in this triple coincidence channel was found to peak at zero, as expected due to momentum conservation, with a full width at half maximum of ±9 au. In the following, we selected this main peak around zero in order to select only *true* coincidence events, where all fragments stem from the same parent molecule. The three-dimensional momentum vectors of all three fragments were then used to calculate the emission angle of the fragments, their kinetic energies, and the total kinetic energy release.

**Figure 2:**
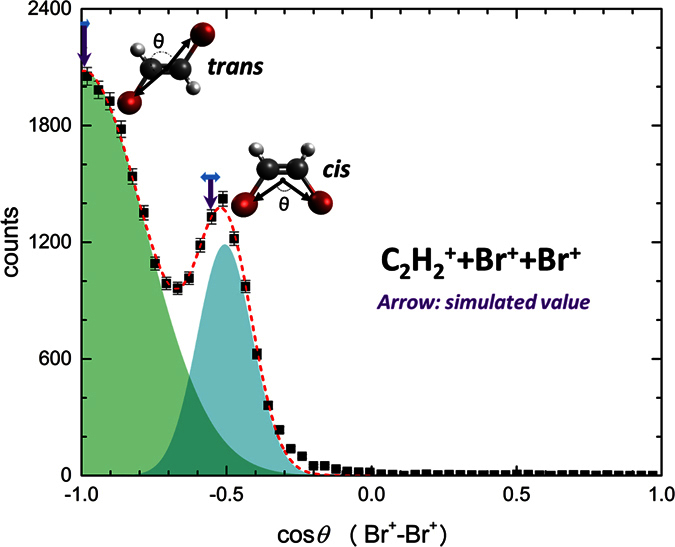
Angle between the Br^+^ ion momenta in the triple-coincidence channel C_2_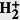 + Br^+^ + Br^+^. A fit of two Gaussians (shaded areas) to the experimental data (black circles) is shown as a red dashed line. The angles expected from a classical Coulomb explosion model are indicated by arrows, with the horizontal bars indicating the broadening due to the experimental momentum resolution.

Figure 2 shows the angle *θ* between the momentum vectors of the two Br^+^ fragments detected in coincidence with a C_2_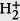 fragment. This angular distribution displays two peaks which we attribute to bromine ions emitted from the *trans* (cos *θ* = −1) and *cis* (cos *θ* = −0.58) isomers. We corroborate this assignment by a numerical Coulomb explosion simulation, assuming purely Coulombic repulsion between point charges located at the center of mass of each fragment and instantaneous creation of the charges followed by explosion from the equilibrium geometry of the neutral molecule. By numerically solving the classical equations of motions for all the fragment ion in the Coulomb field using a 4^th^ order Runge-Kutta method, the momentum vectors and kinetic energies of all fragments can thus be obtained for an ideal Coulomb explosion model. The simulated Br^+^-Br^+^ angles for Coulomb explosion of *cis* and *trans* isomers are shown as purple arrows in Fig. 2 and are in excellent agreement with the two maxima in the experimentally determined distribution. By fitting two Gaussians to the experimental data, we determine a ratio between the *trans* and *cis* isomers of 2.04 ± 0.07, which agrees with the isomeric composition of the sample determined by gas chromatography.

**Figure 3:**
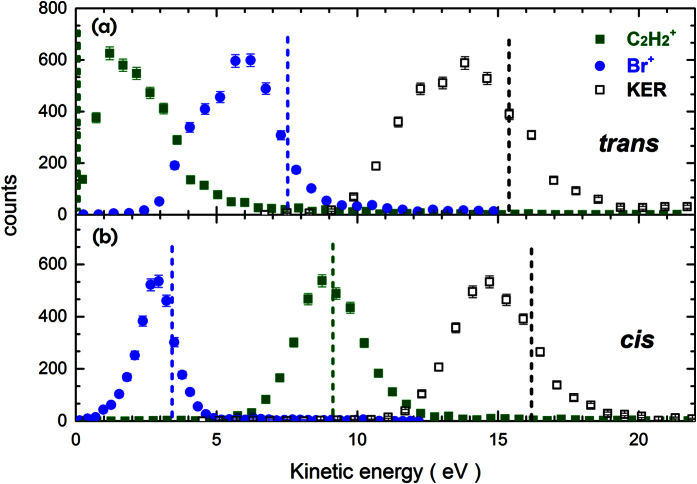
Kinetic energy distributions of the Br^+^ and C_2_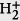 fragments as well as total kinetic energy release in the C_2_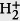 + Br^+^ + Br^+^ triple-coincidence channel for (**a**) *trans* and (**b**) *cis* isomers, as selected by the Br^+^- Br^+^ angle *θ*. The results of the Coulomb explosion model are shown as dashed lines.

**Figure 4:**
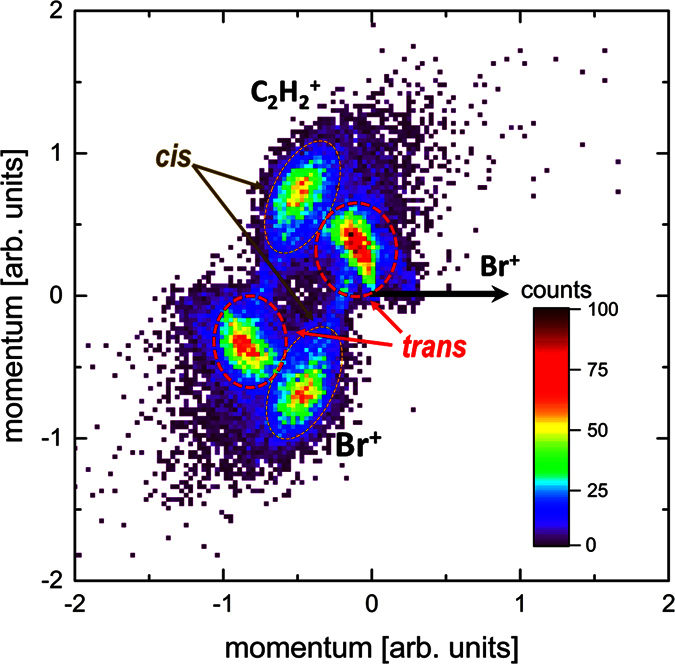
Newton plot of the C_2_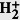 + Br^+^ + Br^+^ triple-coincidence channel. The momenta of C_2_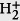 fragments (upper half) and of one of the Br^+^ fragments (lower half) are shown in the frame of the momentum of the second Br^+^ fragment, which is shown as a black horizontal arrow. The momentum vectors of the C_2_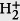 fragment and the first Br^+^ fragment are normalized to the length of momentum vector of the second Br^+^. The contributions corresponding to the *cis* and *trans* isomers are indicated.

By selecting only those coincidence events for which the Br^+^-Br^+^ angles lie within the intervals of cos*θ* = [−0.45, −0.55] and [−0.9, −1.0], respectively, we can experimentally distinguish the *cis* and *trans* isomers and extract the isomer-selected fragmentation kinematics from our data. The isomer-selected kinetic energies of the individual ions and the total kinetic energy release are shown in Fig. 3. For the *trans* isomer, the kinetic energy distribution of the C_2_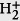-fragment has a maximum around 1 eV and that of the Br^+^-fragments peaks around 6 eV, while for the *cis* isomer, the Br^+^-fragments are emitted with lower and the C_2_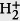-fragment with considerably higher kinetic energy. This can be explained by an “obstructed instantaneous explosion”^28^,^29^ in the case of the *trans* isomer, where the C_2_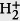-fragment is trapped between the two Br^+^-fragments, while it obtains a much higher kinetic energy in the fragmentation of the *cis* isomer, where it is repelled by the two Br^+^-fragments that are located in the same hemisphere. This simple picture is also corroborated by the results of our Coulomb explosion model calculations shown in Fig. 3, which predict values that lie very close to the maxima of the experimental kinetic energy distribution and also reproduce the difference between the KERs of the two isomers. It can also be visualized directly in a Newton plot^29^ for this particular triple-coincidence channel shown in Fig. 4, where the momenta of two fragments (C_2_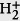 and Br^+^) are shown in the frame of the momentum of the third fragment (Br^+^). For the *trans* isomer, the Newton plot shows that the two Br^+^ fragments are emitted close to back to back, while the C_2_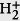 fragment remains almost at rest, as one would expect from an instantaneous fragmentation in the undistorted equilibrium geometry, where the center of mass of the C_2_H_2_ moiety is exactly in the middle between the two Br atoms. The *cis* isomer, however, has a distinctly different fragmentation pattern with C_2_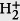 and Br^+^ fragments being emitted at an angle of close to 125 degree with respect to each other and carrying momenta of similar magnitude.

**Figure 5:**
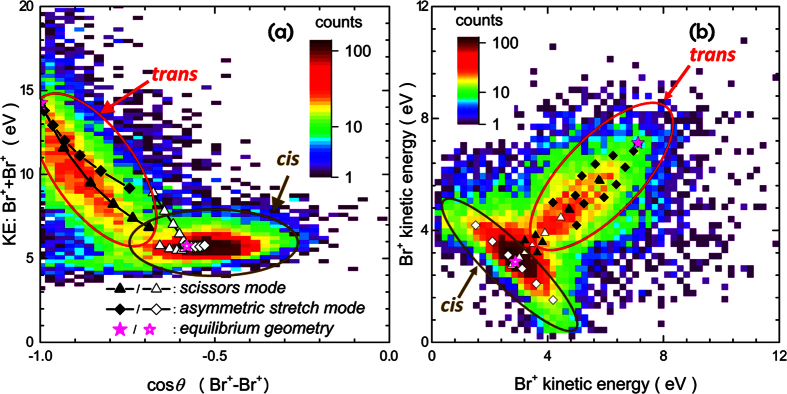
(**a**) Sum of the kinetic energies of the two Br^+^ fragments as a function of the angle *θ* between them, and (**b**) correlation between the kinetic energies of two Br^+^ fragments, both for the C_2_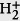 + Br^+^ + Br^+^ triple-coincidence channel. The results of our Coulomb explosion simulations for various stretching and bending modes are shown as symbols, with the stars representing the results for the equilibrium geometries. The plots depict the isomerically mixed sample, but selecting one isomer via selection of the Br^+^ - Br^+^ angle as done for Fig. 3 would select only the contributions shown in the regions marked *cis* and *trans*.

From the shape of the momentum distributions seen in the Newton plots as well as from the good agreement between the experimental data and the simulation, we conclude that the break-up of the molecule into this three-body channel happens mainly via concerted fragmentation in a molecular geometry that still resembles the equilibrium geometry. However, we attribute the widths of the angular distributions and the kinetic energy distributions beyond the experimental momentum resolution to small deviations from this equilibrium geometry due to stretching and bending motions of the molecule in either the neutral molecule (e.g. vibrations due to the temperature of the molecular beam) or in the cationic state(s) that are reached after the ionization. We can include these geometry changes empirically in our Coulomb explosion model by varying the bond lengths and angles is a systematic fashion (see Methods for a sketch of the stretching and bending modes considered here). The results are plotted and compared to the experimental data in Fig. 5. Since the calculations slightly overestimate the kinetic energies, as seen in Fig. 3, the calculated Br^+^ energies are shifted by −0.65 eV for better comparison to the experimental data. In Fig. 5(a), the sum of the kinetic energies of the two Br^+^ fragments is shown as a function of the angle *θ* between the Br^+^ fragments, while Fig. 5(b) shows the correlation between the kinetic energies of the two Br^+^ fragments. In the Coulomb explosion calculations, the C-Br bond distance is varied between 100% and 40% of the equilibrium bond length (in steps of 10%) for the asymmetric stretch mode, and the bond angle by ±25 degrees (in steps of 5 degrees) around the equilibrium angle for bending in the “scissor mode”. A combination of both modes reproduces the shape of the Br^+^-Br^+^ kinetic energy correlation for the *trans* isomer, which is centered around the line of equal energy sharing between the two Br^+^ ions. According to the model calculations, the spread in the angle between the two Br^+^ ions for the *trans* isomer is mainly due to bending in the scissor mode, while the the broadening of the Br^+^ kinetic energy sum and the deviations from equal energy sharing are likely due to asymmetric stretching. For the *cis* isomer, asymmetric stretching of the C-Br bonds reproduces well the almost constant sum of the Br^+^ kinetic energies, but it cannot fully explain the spread in the angle that reaches values up to cos*θ* = −0.2 in the experimental data. This may be an indication that some channels that lead to the fragmentation of the *cis* isomer possibly involve a delayed charging of one of the Br atoms, thus resulting in deviations from the concerted fragmentation model. Further information on the pathways and intermediate ionic states that give rise to these events could be obtained, e.g., from coincident high-resolution Auger electron spectra. Some of the events that are not accounted for in the Coulomb explosion simulation might also stem from a two-step fragmentation mechanism involving intermediate states that are long-lived as compared to the rotational period of the molecule. Such a “delayed fragmentation” usually leads to a ring-like structure in the Newton plots^30^,^31^, which seems to also be present in Fig. 4. Further information about differences in the fragmentation dynamics between the two isomers or possible isomerization dynamics in the cationic state may be obtained from future time-resolved pump-probe experiments.

## Conclusions

Driven by the availability of intense, short-pulse XUV- and soft X-ray sources such as free-electron lasers and high-order harmonic sources, there is a high current interest in developing schemes to directly image molecular structure during ultrafast photochemical reactions. The coincidence Coulomb explosion imaging technique is a promising candidate in this respect, and was used already in a number of time-resolved experiments studying, e.g., the isomerization between acetylene and vinylidene^11^,^12^,^13^,^14^,^15^. Here, we have shown that this technique is also applicable to (single-photon) XUV-induced inner-shell ionization and can be used to distinguish geometric isomers and determine their isomer ratio. In particular, our results demonstrate that the Coulomb explosion of 1,2-dibromoethene into C_2_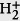 + Br^+^ + Br^+^ after Br(3d) inner-shell ionization can be used as a probe for the initial geometric structure of the molecule, enabling us to experimentally distinguish the *cis* and *trans* isomers and to measure the isomer-specific ionization and fragmentation dynamics of a mixed sample. While some isomers can be easily separated due to their different physical properties, others cannot be easily prepared as pure samples, e.g. in cases where they can interconvert between different structures at room temperature. The coincidence Coulomb explosion imaging method allows studying isomer-specific properties also in the latter case. More importantly, this imaging technique provides an *in-situ* method to quantify the isomer ratio, thus opening up new possibilities for pump-probe experiments studying ultrafast isomerization reactions using optical laser, free-electron lasers, or high-harmonic generation sources.

## Methods

### Experimental Details

In order to measure ions and electrons in coincidence, we used a double-sided velocity map imaging (VMI) setup, as shown above in Fig. 1(a). The detection scheme is different from a conventional VMI system^32^ in the sense that microchannel plate detectors equipped with multi-hit delay-line anodes (RoentDek DLD80 for the electrons and RoentDek HEX80 for the ions) are used to record both position and time information for the charged fragments^33^,^34^,^35^. In addition, the spectrometer consists of *four* electrostatic lenses that simultaneously focus *both* electrons and ions onto the two detectors at opposite sides of the spectrometer. The lens voltages chosen in this experiment enabled the collection of electrons up to 150 eV and singly charged ions up to 15 eV, over the full solid angle.

The experiments were performed at beamline 10.0.1.3 of the Advanced Light Source (ALS) at Lawrence Berkeley National Laboratory during the standard ALS multi-bunch top-off mode of operation. The data acquisition system, consisting of two multi-hit time-to-digital (TDC) converters, was triggered by the detection of an electron, which arrived at the detector after a flight time of approximately 5 nanoseconds. The time of flight of the ions was then measured with respect to the arrival time of the prompt electrons. The position and time-of-flight information of the ions are used to reconstruct their full three-dimensional momentum vectors. Since the electric field in the spectrometer is not homogeneous, we cannot derive analytical formulas for this reconstruction but use the SIMION software package^27^ to simulate the expected time of flight and hit positions for ions starting in the interaction region with different kinetic energies and emission angles. We then fit a polynomial to the simulated data to construct empirical formulas connecting the hit position with the momentum vector components parallel to the detector, P_*x*_ and P_*v*_, and the time-of-flight spread of each ion with the momentum component along spectrometer axis, P_*z*_.

The chemical sample used in the experiment was commercially purchased (Sigma Aldrich, 98.3% purity) and contained a mixture of *cis* and *trans* isomers at a mixing ratio of 32.1% (*cis*) to 66.2% (*trans*), according to a gas chromatography analysis by the vendor. The sample is liquid at room temperature and was brought into the gas phase via supersonic expansion through a 30 micron aperture. The vapor pressure of C_2_H_2_Br_2_ is 31 mmHg at 25 C^36^, which was sufficient to form a molecular beam without additional carrier gas. After passing through a skimmer with a 500 micron diameter, the molecular beam is crossed by a beam of linearly polarized ALS photons in the interaction center of the VMI spectrometer.

### Coulomb explosion calculations

**Figure 6:**
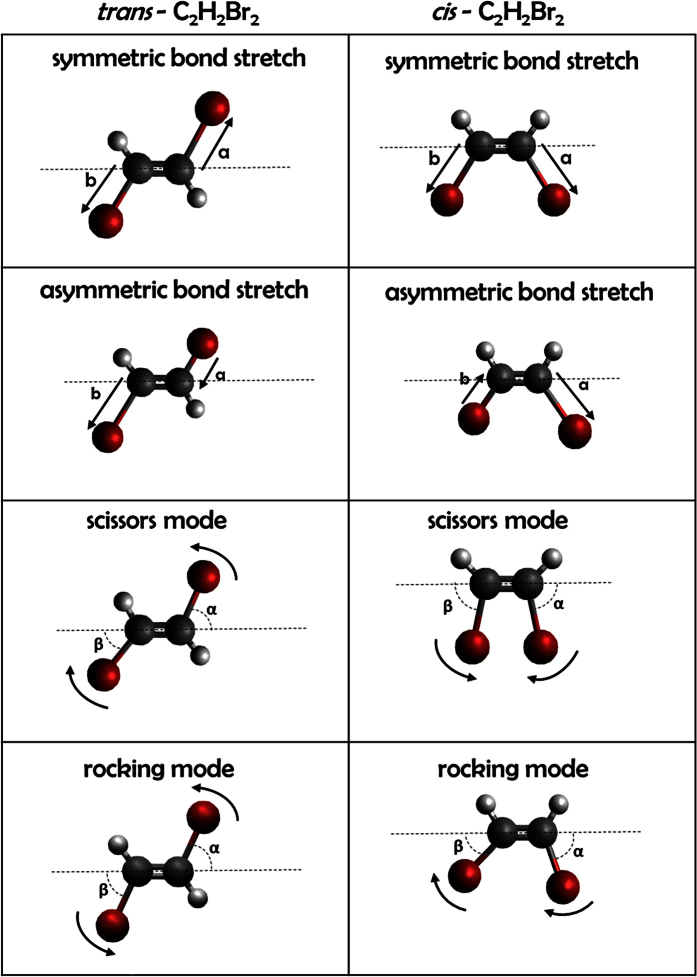
Overview of stretching and bending modes in *trans* and *cis* C_2_H_2_Br_2_ considered in our Coulomb explosion simulations. The effect of bending in the “rocking mode” (i.e. both Br atoms rotating in the same direction) is not shown in Fig. 5 since it barely influences the outcome of our model calculations, while symmetric stretching of the C-Br bonds simply broaden the kinetic energy distribution at a fixed value of cos*θ*.

For the Coulomb explosion calculations discussed above, we have used the equilibrium geometry of the neutral *trans* and *cis* dibromoethelene (C_2_H_2_Br_2_) molecule as determined by the Gaussian software package^37^. However, it is known that the molecule may undergo geometry changes, e.g. due to vibrations in the neutral or cationic states or due to a geometric rearrangement, such as the beginning of an isomerization, that may occur during the photoionization process *prior* to the Coulomb break-up. In order to model the effect of these deviations from the equilibrium geometry in our Coulomb explosion simulations, we have broken down the large number of vibrational modes and other possible geometry changes into a few basic stretching and bending motions, as shown in Fig. 6, that we can systematically include in our calculations. We have limited our selection to two stretching and two bending modes involving the C-Br bonds that we considered to be the most likely and most relevant for our case, but note that this is only a subset of all possible modes that can be considered. Within our classical calculations, we cannot distinguish if the geometric changes occur in the neutral or cationic state and we cannot determine the cause of these changes, but this information may be derived from more detailed calculations of the electronic structure and dynamics or from future time-resolved experiments.

## Additional Information

**How to cite this article**: Ablikim, U. *et al*. Identification of absolute geometries of *cis* and *trans* molecular isomers by Coulomb Explosion Imaging. *Sci. Rep.* **6**, 38202; doi: 10.1038/srep38202 (2016).

**Publisher's note:** Springer Nature remains neutral with regard to jurisdictional claims in published maps and institutional affiliations.
